# Role of [18F]FMISO PET Imaging for the Evaluation of Gliomas: A Comprehensive Literature Review

**DOI:** 10.3390/diagnostics16091284

**Published:** 2026-04-24

**Authors:** Francesco Dondi, Pietro Bellini, Alberto Miceli, Silvia Lucchini, Gian Luca Viganò, Roberto Rinaldi, Luca Camoni, Michela Cossandi, Alfredo Muni, Francesco Bertagna

**Affiliations:** 1 Nuclear Medicine, ASST Spedali Civili di Brescia and University of Brescia, 25123 Brescia, Italy; francesco.dondi@unibs.it (F.D.);; 2 Nuclear Medicine, ASST Spedali Civili di Brescia, 25123 Brescia, Italy; pietro.bellini@asst-spedalicivili.it (P.B.); silvia.lucchini@asst-spedalicivili.it (S.L.); roberto.rinaldi@asst-spedalicivili.it (R.R.); michela.cossandi@asst-spedalicivili.it (M.C.); 3 Department of Nuclear Medicine, Azienda Ospedaliero-Universitaria SS. Antonio e Biagio e Cesare Arrigo, 15100 Alessandria, Italy; amuni@ospedale.al.it; 4 Clinical Engineering, ASST Spedali Civili di Brescia, 25123 Brescia, Italy; gianluca.vigano@asst-spedalicivili.it; 5 Allied Health and Social Care Professions Directorate, ASST Spedali Civili di Brescia, 25123 Brescia, Italy; luca.camoni@asst-spedalicivili.it

**Keywords:** positron emission tomography, PET, glioma, glioblastoma, [18F]FMISO, [18F]fluoromisonidazole

## Abstract

[18F]fluoromisonidazole ([18F]FMISO) positron emission tomography (PET) imaging has been explored for its potential use as an added modality for the assessment of gliomas. The aim of this review was to synthesize the existing literature investigating this topic. A comprehensive search of the PubMed/MEDLINE, Scopus, and Embase databases was performed to identify eligible original research investigating the role of [18F]FMISO PET imaging for the evaluation of gliomas. Thirty-tree studies were included in the present review. Overall, the findings reported suggest that [18F]FMISO PET may have relevant clinical implications in glioma assessment and management, including improved tumor characterization, differential diagnosis between different grades and other neoplasms, prognostic stratification, and potential guidance for personalized therapeutic strategies. The available evidence supports the idea that [18F]FMISO PET can act as a robust and biologically meaningful imaging modality in gliomas. Its strengths lie in tumor grading, prognostication, and treatment monitoring, particularly in glioblastoma. Future research should focus on standardized imaging protocols, integration with molecular classification frameworks, and validation of hypoxia-guided therapeutic strategies.

## 1. Introduction

Gliomas represent the most common primary tumors of the central nervous system, arising from glial cells and encompassing a biologically and clinically heterogeneous spectrum of neoplasms. According to the current World Health Organization (WHO) classification, gliomas are stratified based on both histopathological and molecular features, reflecting advances in the understanding of tumor biology and genetic alterations [1]. This group includes low-grade gliomas (LGGs) as well as highly aggressive entities such as glioblastomas, which remain associated with a dismal prognosis despite multimodal treatment [2,3,4]. The infiltrative growth pattern, marked intratumoral heterogeneity, and dynamic microenvironmental changes contribute to therapeutic resistance and inevitable recurrence in high-grade disease [5,6,7,8,9,10]. The management of gliomas consists of surgical approaches with a subsequent chemo- or radiotherapy regimen [11,12]. Consequently, accurate imaging assessment at diagnosis, during treatment planning, and throughout follow-up is crucial to guide clinical decision-making and improve patient management [13,14,15]. In this setting, magnetic resonance imaging (MRI) is the standard modality for glioma evaluation, providing detailed anatomical information and advanced functional parameters, such as perfusion and diffusion metrics, which support the identification of aggressive forms, and which is also able to differentiate tumor progression from treatment-related effects, including pseudoprogression and radiation necrosis [16,17,18,19,20,21].

Positron emission tomography (PET) imaging may be employed for the evaluation of these neoplasms, particularly through radiolabeled amino acid tracers, even though several other radiopharmaceuticals have been investigated for this purpose [17,22,23,24,25,26,27]. Worldwide, the tracer mostly used to perform PET imaging is [18F]fluorodesoxyglucose ([18F]FDG), given its ability to reflect the glycolytic activity of the tissues analyzed in the scan and, based on this property, this radiopharmaceutical has demonstrated its usefulness in evaluating a broad spectrum of pathological conditions, including both neoplastic and benign diseases [28,29,30]. Over the past decades, several PET radiotracers have been investigated in neuro-oncology and radiolabeled amino acids such as [11C]methionine ([11C]MET) and [18F]fluoroethyl-L-tyrosine ([18F]FET) have demonstrated superior tumor-to-background contrast compared with [18F]FDG, improving lesion delineation, biopsy targeting, radiotherapy planning, and response assessment (Table 1) [31,32,33]. Several other radiopharmaceuticals have been used for glioma PET imaging. These tracers highlight the added value of molecular imaging with different radiopharmaceuticals in characterizing glioma biology beyond anatomical imaging alone.

**Table 1:** List of the main PET tracers that have been employed for glioma imaging.

Radiopharmaceutical	Mechanism of Uptake
[11C]MET	Protein synthesis
[18F]FET	Amino acid metabolism
[18F]FDG	Glycolysis
[18F]PSMA-1007	Receptor binding
[18F]fluciclovine	Amino acid metabolism
[18F]DOPA	Amino acid uptake and dopamine synthesis
[11C]choline	Membrane replication
[18F]FLT	Protein synthesis
[68Ga]FAPI	Receptor binding

[11C]MET: [11C]methionine; [18F]FET: [18F]fluoroethyl-L-tyrosine; [18F]FDG: [18F]fluorodesoxyglucose; [18F]PSMA-1007: [18F]prostate-specific membrane antigen; [18F]DOPA: 6-[18F]fluoro-L-3,4-dihydroxyphenylalanine; [18F]FLT: 3′-deoxy-3′-[18F]fluorothymidine; [68Ga]FAPI: [68Ga]fibroblast activation protein inhibitor.

Among the key biological processes driving glioma aggressiveness, tumor hypoxia has gained increasing attention. Hypoxia is a hallmark of rapidly proliferating solid tumors and is particularly relevant in high-grade gliomas (HGGs) [34,35,36]. It may contribute to angiogenesis, metabolic reprogramming, genomic instability, and selection of more aggressive cellular clones. Importantly, hypoxic tumor regions are associated with resistance to radiotherapy—due to the reduced generation of oxygen-dependent free radicals—and decreased sensitivity to certain chemotherapeutic agents [34,35,36,37,38,39]. Therefore, the ability to noninvasively assess and spatially map hypoxia may provide critical prognostic information and support personalized therapeutic strategies. Hypoxia-specific PET radiotracers have been developed to selectively accumulate in viable cells under low-oxygen conditions [40,41]. Among these, [18F]fluoromisonidazole ([18F]FMISO) is one of the most extensively studied agents, as it is a nitroimidazole compound that undergoes intracellular reduction and becomes irreversibly trapped in hypoxic cells, allowing visualization and quantification of hypoxic tumor subvolumes [42,43,44]. In gliomas, [18F]FMISO PET has been explored for its potential role in identifying biologically aggressive regions, predicting treatment response, guiding radiotherapy dose escalation and monitoring changes in tumor oxygenation during therapy [45,46,47]. Other hypoxia-related radiopharmaceuticals have been used in the past to evaluate gliomas, though with limited reporting. [18F]fluoroazomycinarabinoside ([18F]FAZA) and [64Cu]diacetyl-bis(N4-methylthiosemicarbazone) ([64Cu]ATSM) also allow visualization of tumor hypoxia and, although promising, their clinical use in glioma is still limited by variability in uptake patterns and less extensive validation when compared with established tracers. Initial evidence of their possible role in prognosis assessment, evaluation of response to therapy, and prediction of grade, in particular in HGGs, have been reported, however [48,49,50,51,52].

In this context, the present article aims to provide an in-depth overview of the role of [18F]FMISO PET in the evaluation of gliomas by summarizing the current available literature.

## 2. Materials and Methods

A comprehensive literature search was performed across three databases (PubMed/MEDLINE, Scopus, and Embase) to identify studies evaluating the role of [18F]FMISO PET imaging for the assessment of gliomas. The search strategy included the following terms: (“PET” OR “positron emission tomography” OR “PET/CT”) AND (“FMISO” OR “fluoromisonidazole”) AND (“glioma” OR “glioblastoma”).

Data extraction was performed and general study characteristics were collected for each article, including the first author’s name, publication year, country of origin, study design, radiopharmaceuticals employed, and size of the cohort. In addition, technical and imaging-related information was retrieved, such as the type of PET system used, the administered activity of the radiotracers, and the main quantitative and qualitative outcomes reported by the authors. The principal findings of the studies included in this review are summarized in Section 3.

## 3. Results

Different papers have assessed the value of [18F]FMISO PET imaging for the evaluation of gliomas and have focused particular attention on its ability to evaluate the presence of disease or its relapse, the differential diagnosis with other neoplasms, the different diagnosis between HGG and LGG, the correlation with biomarkers, and its prognostic value, and have also compared this modality with other PET imaging or other imaging modalities [53,54,55,56,57,58,59,60,61,62,63,64,65,66,67,68,69,70,71,72,73,74,75,76,77,78,79,80,81,82,83,84,85].

Seventeen studies included in the review had a retrospective design [53,54,55,57,58,59,62,63,66,72,73,77,78,80,82,84,85], while 16 were prospective [56,60,61,64,65,67,68,69,70,71,74,75,76,79,81,83]. Twenty-one studies were performed using PET/CT tomographs [62,63,65,66,67,68,70,71,72,73,74,75,76,77,78,80,81,82,83,84,85], 9 with PET systems [53,54,55,56,57,58,59,60,69], 2 with PET/MR [64,79] while 1 included both PET and PET/CT imaging [61]. Regarding the radiopharmaceuticals employed, 21 studies were performed using only [18F]FMISO [53,57,58,59,62,63,64,65,66,67,68,69,70,71,73,74,76,79,80,81,84], 7 with both [18F]FMISO and [18F]FDG [55,56,61,77,78,82,85], 2 with [18F]FMISO and [18F]FLT [75,83], 1 with [18F]FMISO and [15O]H_2_O [54], 1 with both [18F]FMISO and [11C]MET [60], and finally, one paper used [18F]FMISO, [18F]FDG, [11C]MET and [18F]FLT [72]. The main characteristics of the included studies and their principal findings are summarized in Table 2 and Table 3 and in the Supplementary Materials.

### 3.1. Assessment of Gliomas

Different papers underlined the ability of [18F]FMISO imaging to reveal the presence of gliomas or glioblastomas that were characterized by different tumor-to-plasma ratios and a higher constant of uptake (K1) compared with normal brain [53,54]. Further analyses investigated the added value of this imaging modality to differentiate between gliomas and other neoplastic conditions, revealing, for example, higher K1 and volume of tracer distribution compared with meningioma [54]. Interestingly, it has been reported that tumor-to-normal brain ratio (TNR) was significantly higher for glioblastoma compared with primary central nervous system lymphoma (PCNSL) (*p* < 0.001) with an area under the curve (AUC) value of 0.833, higher than that of [18F]FDG TNR (0.825); the combination of both imaging modalities, however, reached the highest AUC (0.900) [78].

### 3.2. Differential Diagnosis Between Different Gliomas

One of the main fields of application of [18F]FMISO PET imaging was the differential diagnosis between gliomas with different grading and, in general, tracer uptake was observed in all HGG (grades III and IV) but not in LGG (grades I and II) [56,62]. Interestingly, in different papers higher tumor-to-blood ratio (TBR) and hipoxic volume (HV) for grade IV gliomas compared with grade III neoplasms were reported [62,63]. In general glioblastomas were characterized by higher uptake compared with other gliomas [72]. In this setting, it has been reported that uptake in the tumor was greater than that in the surrounding brain tissues, whereas all non-glioblastoma neoplasms had uptake in the tumor equal to that in the surrounding brain tissues (*p* ≤ 0.001), with sensitivity and specificity of 100% and 100% respectively for the diagnosis of glioblastoma compared with values of 100 and 66% for [18F]FDG, respectively [61,67]. Moreover, higher tumor-to-cerebellum ratio and uptake volume were reported for glioblastomas compared with non-glioblastomas lesions (*p* ≤ 0.001 in both cases) [61].

### 3.3. Comparison Between [18F]FMISO and Other Radiopharmaceuticals

The comparison between [18F]FMISO and other radiotracers has also been explored in different manuscripts. In this setting, positive correlation between [18F]FMISO tumor uptake and perfusion assessed with [15O]H_2_O in the first minutes after injection have been reported. However, the two were independent at late acquisition, suggesting that late [18]F-FMISO images provide a spatial description of hypoxia in brain tumors that is independent of blood–brain barrier (BBB) disruption and tumor perfusion and that hypoxia in these tumors may develop irrespective of the magnitude of perfusion [54]. When focusing on [18F]FDG, it has been reported that there is a positive but not significant correlation between the uptake of the two tracers in glioblastoma, that was, however, different when compared with other neoplasms [55]. Moreover, a correlation between the two imaging modalities within individual tumor grades has been reported, with hypometabolic gliomas not showing [18F]FMISO uptake, although the inverse may be true, and with areas of marked [18F]FMISO uptake often corresponding to areas of only slightly increased [18F]FDG uptake [56]. Interestingly, a single study has reported that the viable hypoxic tissue assessed by [18F]FMISO PET is related to the tumor aggressiveness evaluated by [11C]MET PET in newly diagnosed glioblastoma, with a strong correlation between the tumor volume visualized with the two modalities (*p* < 0.01) [60]. When combining both [18F]FLT and [18F]FMISO imaging, it has been demonstrated that all glioblastomas exhibit heterogeneous regions with uptake of both tracers, even with hotspots outside the enhanced volume at MRI. This is particularly so for higher proliferative regions assessed by [18F]FLT, while hypoxic uptake was mainly visualized within the enhancing region [75]. Moreover, the combination of dual [18F]FMISO and [18F]FLT PET imaging was able to provide a detailed characterization of tumor microenvironment in glioblastoma and WHO grade 4 astrocytomas, revealing an inverse correlation between lesion perfusion and hypoxia [83]. Lastly, Miyake et al. [72] have suggested that the combined administration of four different positron emitter radiopharmaceuticals ([18F]FMISO, [18F]FDG, [11C]MET, and [18F]FLT) might aid in the preoperative differential diagnosis of gliomas according to the 2016 WHO criteria since neoplasms with different grading were characterized by diverse uptakes.

### 3.4. Comparison Between [18F]FMISO and MRI

When comparing MRI and [18F]FMISO PET findings, it has been reported that hypoxia may drive the peripheral growth of glioblastomas, supporting the idea of the spatial link between the volumes and surface areas of hypoxic and MRI regions [58]. Furthermore, it has been suggested that the viable hypoxic tissue is related to the neovascularization in gadolinium-enhanced MRI in newly diagnosed glioblastoma [60]. Similarly, as high hypervascularization assessed with MRI was found to be significantly correlated with hypoxia (*p* < 0.001), the hypothesis of a tight association between hypoxia and angiogenesis has been suggested [68]. Partially in contradiction, limited correlations between the two imaging modalities with poor spatial correspondence have been reported by Preibisch et al. [69], suggesting that the two methods appear to provide complementary rather than redundant information about HGG biology.

### 3.5. Correlation with Biomarkers

Different papers have assessed the correlation between [18F]FMISO PET imaging and the presence of several glioma biomarkers. In this setting, a significant relationship has been reported between tracer uptake and the expression of vascular endothelial growth factor receptor 1 (VEGF-R1) and Ki67 [56,63,67]. Interestingly, a correlation between standardized uptake value (SUV) and VEGFR has been demonstrated in newly diagnosed glioma, but not in recurrent neoplasms [63]. Expression of hypoxia biomarkers (CAIX, HIF-1α) and angiogenesis markers (VEGF, Ang2, rCBV) were found to be significantly higher in gliomas with [18F]FMISO uptake and correlations between the degree of hypoxia [hypoxic volume (HV) and SUVmax] and expression of HIF-1α, CAIX, VEGF, Ang2, and rCBV (*p* < 0.01 for all) have been reported [67]. Moreover, mRNA expression of glucose-6-phosphatase 3 (G6PC3) was found to be significantly higher in glioblastomas with high [18F]FMISO uptake compared with those with low uptake (*p* < 0.001), suggesting that G6PC3 expression might be facilitated by hypoxic conditions in glioblastomas, resulting in a high uptake of both hypoxic radiotracer and [18F]FDG [82]. Interestingly, correlations between hypoxia-related parameters and net rate of cell proliferation (*p* < 0.04) and biological aggressiveness (*p* < 0.001) have been demonstrated [59]. Focusing on isocitrate dehydrogenase (IDH) mutation, a significantly higher uptake of [18F]FMISO has been reported in IDH-wildtype tumors when compared with IDH-mutant newly diagnosed gliomas; however, in WHO grade III anaplastic astrocytomas or oligodendrogliomas, no significant differences were confirmed between the two groups [77,81]. In this setting, [18F]FMISO PET imaging might provide a valuable tool for differentiating between the IDH mutation status of 2021 WHO classification grade III and IV adult-type diffuse gliomas [81]. Lastly, it has been suggested that [18F]FMISO uptake within a lesion indicates the presence of histological micro-necrosis [84].

### 3.6. Prognosis and Therapy Response

One of the main fields of research for the application of [18F]FMISO PET imaging in gliomas is its prognostic value in both the pre- and post-treatment settings, suggesting that the higher quote of dying subjects had [18F]FMISO positive neoplasms [56]. In general, it has been reported that patients with low tracer uptake, even considering HV and TNR ratio, were characterized by a longer overall survival (OS) time when compared with positive subjects, and in some cases these parameters were reported as significant independent predictors [57,58,63,65,67,73,76]. In other cases, SUVpeak was an independent predictor of outcome [73]. An interesting paper by Yamaguchi et al. [66] has demonstrated that MRI-[18F]FMISO double responders showed significantly longer OS, whereas no OS difference between MRI-only responders and non-responders were reported, suggesting that high TNR uptake is a significant independent prognosticator. Toyonaga et al. [85] have demonstrated that, when using both [18F]FDG and [18F]FMISO imaging, hMTV (the volume of [18F]FMISO/[18F]FDG double-positive) and hTLG (the product between hMTV and [18F]FDG SUVmean) were independent factors affecting both progression-free survival (PFS) and OS. Muzi et al. [73] performed a prognostic study to assess the added value of [18F]FMISO-based radiomics analysis in the evaluation of OS, revealing that the model selection that included radiomic features showed the additional prognostic value for OS of specific higher order texture features, leading to an increase in relative risk prediction performance by a further 5%. Moreover, patients with shorter PFS had higher HV and TNR ratios, with those parameters also reaching significance in multivariable analyses in different paper [57,65]. In this setting, it has been reported that PFS decreased significantly with HV (*p* 0.009) [76].

Two studies have evaluated the role of [18F]FMISO PET imaging for the assessment of response to bevacizumab therapy in HGG, revealing a reduction in HV concurrent with therapy in all patients with recurrent tumor and the absence of hypoxia-related uptake in the case of disease progression [64]. Similarly, in the case of neoadjuvant glioblastomas, therapy decrease in [18F]FMISO uptake was demonstrated and subsequent recurrence was underlined by the presence of radiopharmaceuticals accumulation, suggesting that hypoxia PET might be useful for monitoring the duration of therapy efficacy by reflecting tumor oxygenation [80]. In this scenario, it has also been reported that the presence of pseudoprogression was retrospectively found to be associated with the absence of hypoxia, that areas of pseudoprogression were characterized by a lower mean hypoxic fraction compared with recurrence, and that [18F]FMISO PET imaging, in particular PET/MRI, may serve as a biologically specific metric of therapeutic failure [64,79].

### 3.7. Miscellanea

Interestingly, some papers focused their attention on different modalities and approaches to generate [18F]FMISO PET images, reporting differences in defining the HV within a tumor using different TBR [71]. Additionally, it has been reported that TBR produced broader images of the tumor than TNR given the same thresholds on intensity and given that weak to moderate correlation coefficients with spectral analysis hypoxia were found for most thresholding values and imaging times [71]. Interestingly, a scan time of 90 min after radiotracer injection has been suggested as suitable for a single scan acquisition irrespective of tumor status being highly hypoxic or perfused [70]. Moreover, it has been suggested that compartmental modeling and spectral analysis methods based on dynamic PET imaging equally located tumor hypoxia, suggesting that the combination of these approaches was found to be more appropriate in generating voxel-based hypoxia images [74].

## 4. Discussion

The present body of evidence highlights a possible significant role of [18F]FMISO PET imaging in the assessment of gliomas by characterizing tumor hypoxia. Across studies, [18F]FMISO demonstrated the ability to distinguish neoplastic tissue from normal brain parenchyma based on higher neoplasm uptake [53,54,78]. These findings confirm that hypoxia-targeted PET imaging may provide functional information that complements conventional structural imaging and reflects specific biological features of tumor aggressiveness. In this setting, it should be noted that the use of [18F]FMISO is relatively uncommon in glioma imaging, primarily due to its low tumor-to-tissue contrast [52]. This limitation reduces image clarity and diagnostic efficiency, making it less favorable when compared with other tracers. As a consequence, its application typically requires prolonged PET imaging protocols, with data acquisition occurring 2 to 4 h after tracer injection, which can be less practical in clinical settings [52].

A major area of application concerns the differential diagnosis among glioma subtypes, HGG and LGG, and between gliomas and other intracranial neoplasms. Higher TNR and uptake parameters in glioblastoma compared with PCNSL or meningioma suggest that [18F]FMISO may contribute to the improvement of diagnostic accuracy [54,78]. Notably, the reported data indicate that [18F]FMISO performs at least comparably to, and in some cases slightly better than, [18F]FDG in differentiating glioblastoma from other entities [61,67,72]. Importantly, the combination of hypoxia and metabolic imaging achieved the highest diagnostic accuracy, underscoring the complementary nature of these tracers. With regard to tumor grading, the absence of significant tracer uptake in LGG and its consistent presence in HGG supports the idea of a strong association between hypoxia and tumor malignancy [56,62]. The observation of higher TBR ratios and HVs in grade IV tumors compared with grade III lesions further reinforces the link between increasing hypoxic burden and biological aggressiveness. In particular, glioblastoma has demonstrated higher uptake intensity and larger hypoxic volumes when compared with non-glioblastoma neoplasms, with excellent reported sensitivity and specificity for diagnosis in selected cohorts [61,62,63,67,72]. These findings seem to be in line with the idea that hypoxia may promote angiogenesis, genomic instability, and resistance to therapy in HGG [34,35,86,87].

The comparison between [18F]FMISO and other PET tracers provides further insight into tumor pathophysiology. Early correlations between [18F]FMISO uptake and perfusion assessed with [15O]H_2_O, followed by independence at later acquisition times, support the concept that delayed [18F]FMISO imaging may reflect real cellular hypoxia rather than perfusion or BBB disruption [54]. The generally weak or non-significant correlation with [18F]FDG uptake may suggest that glucose metabolism and hypoxia represent partially overlapping but distinct biological processes [55,56]. Similarly, the association between HV assessed by [18F]FMISO and tumor aggressiveness evaluated with amino acid tracers, such as [11C]MET, indicates that multiparametric PET approaches may provide a more comprehensive characterization of tumor biology [60,75,83]. The integration of [18F]FMISO with proliferation imaging (e.g., [18F]FLT) further emphasizes intratumoral heterogeneity. The spatial mismatch between hypoxic and highly proliferative regions, including hotspots outside MRI contrast-enhanced areas, supports the concept that glioblastoma is characterized by a complex microenvironment in which hypoxia, proliferation, and perfusion are dynamically interconnected [75,83]. These findings may have implications for radiotherapy planning and targeted therapeutic strategies, particularly in the context of biologically guided dose escalation [37].

In light of current evidence, the relationship between [18F]FMISO uptake and MRI-derived parameters remains partially controversial. While several studies have demonstrated correlations between hypoxia and neovascularization or hypervascularization on gadolinium-enhanced MRI—suggesting a tight link between hypoxia and angiogenesis—others have reported limited spatial correspondence [58,60,68,69]. This apparent discrepancy likely reflects the fact that MRI and hypoxia PET probe different biological aspects of tumor physiology. In particular, [18F]FMISO PET directly reflects cellular hypoxia, whereas contrast-enhanced MRI provides indirect measures of vascular permeability and blood–brain barrier disruption, which are influenced by multiple factors beyond angiogenesis alone. Moreover, spatial and temporal heterogeneity of gliomas, together with methodological differences across studies (e.g., image resolution, registration accuracy, and threshold definitions), may further contribute to inconsistent findings. Therefore, rather than being redundant, these modalities should be considered complementary tools that together provide a more complete depiction of glioma biology.

Correlations between [18F]FMISO uptake and molecular biomarkers further strengthen its biological validity. Significant associations with angiogenic markers (e.g., VEGF-related pathways), proliferation indices (Ki-67), and hypoxia-regulated proteins (HIF-1α, CAIX) indicate that the uptake of this radiotracer mirrors the molecular adaptations to hypoxic stress [56,59,63]. The observed differences in uptake according to IDH mutation status are particularly relevant in the era of molecular classification. Higher uptake in IDH-wildtype tumors compared with IDH-mutant gliomas aligns with the more aggressive clinical behavior of IDH-wildtype disease and suggests a potential role for [18F]FMISO PET in non-invasive molecular stratification [77,81,84]. In this regard, PET imaging may be helpful for personalized therapy and precise medicine in patients with specific biomarkers [88,89,90].

One of the most clinically relevant findings across studies is the prognostic value of hypoxia imaging. Patients with lower tracer uptake, smaller HVs, and lower TNR demonstrated longer OS and PFS. Moreover, in several analyses, hypoxia-related parameters emerged as independent prognostic factors [56,57,58,63,65,66,67,73,76,85]. Furthermore, though they are embryonic, some insight has suggested that advanced radiomic approaches appear to further enhance risk stratification by capturing spatial heterogeneity beyond conventional SUV metrics [73]. These findings seem to reinforce the concept that tumor hypoxia is not merely a descriptive biomarker but may also have a determinant role in the outcome of patients. In the therapeutic setting, preliminary evidence suggests that [18F]FMISO PET may serve as a valuable tool for monitoring response to antiangiogenic therapy such as bevacizumab [64,79,80]. The observed reduction in HV during treatment and its reappearance at recurrence indicate that hypoxia imaging may reflect dynamic changes in tumor oxygenation. Furthermore, the association between the absence of hypoxia and pseudoprogression highlights the potential role of [18F]FMISO PET, especially in hybrid PET/MRI systems, in distinguishing treatment-related changes from true tumor progression [80].

Finally, methodological aspects deserve consideration. Variability in HV definition according to different TBR or TNR thresholds underscores the need for standardization [71]. The suggestion that imaging at approximately 90 min post-injection provides reliable hypoxia assessment may contribute to protocol harmonization. Additionally, the comparable performance of compartmental modeling and spectral analysis for dynamic imaging indicates that advanced quantitative approaches may improve voxel-wise characterization of tumor hypoxia [70,71,74].

Overall, the findings reported in this review suggest that [18F]FMISO PET may have clinical implications in glioma management, including improved tumor characterization, prognostic stratification, and potential guidance for personalized therapeutic strategies. Moreover, hypoxia imaging may support treatment monitoring and help differentiate tumor progression from therapy-related changes. Future research should focus on prospective multicenter studies to validate these findings across larger and more heterogeneous patient populations, standardize imaging acquisition and analysis protocols, and reduce methodological variability between centers. In addition, further work should explore the integration of [18F]FMISO PET with multiparametric MRI, other complementary PET tracers, and advanced radiomics or machine learning approaches, in order to better capture tumor heterogeneity and improve the non-invasive characterization of glioma biology. Such integrated strategies may ultimately enhance diagnostic accuracy, enable more precise treatment planning, and support improved patient stratification in both clinical and research settings. Future research should focus on prospective multicenter studies to validate these findings across larger and more heterogeneous patient populations, standardize imaging acquisition and analysis protocols, and reduce methodological variability between centers. In addition, further work should explore the integration of [18F]FMISO PET with multiparametric MRI, other complementary PET tracers, and advanced radiomics or machine learning approaches, in order to better capture tumor heterogeneity and improve the non-invasive characterization of glioma biology. Such integrated strategies may ultimately enhance diagnostic accuracy, enable more precise treatment planning, and support improved patient stratification in both clinical and research settings. Moreover, longitudinal studies are needed to better understand how these imaging biomarkers evolve over time, particularly in response to therapy, and whether they can reliably capture early treatment-induced changes before anatomical alterations become evident. Correlating imaging features with molecular and genetic tumor profiles (e.g., IDH mutation status, MGMT methylation, and hypoxia-related gene expression) could further strengthen the biological interpretation of imaging signals. Finally, the development of standardized quantitative thresholds and robust validation frameworks will be essential to facilitate translation into routine clinical practice and to ensure reproducibility across institutions. Several limitations related to the characteristics of the studies included in this review may influence the findings here presented. First, most studies involved small and heterogeneous patient cohorts. Furthermore, though specific analyses were performed, some studies included both LGG and HGG. As a result, the descriptive analyses conducted rely on relatively limited samples. In addition, the majority of the included papers had a retrospective design. Moreover, the studies included in the review span a period of more than 30 years, meaning that different technologies, tomographs, image acquisition and processing methods were used, potentially influencing the generalizability of the findings presented. The current level of evidence consists mostly of single-center, retrospective and small-sample studies with heterogeneity in study designs, patient populations, and analytical methods that limits definitive conclusions. In this setting, across the studies included in the review, the prognostic value of [18F]FMISO PET imaging seems to be the parameter that has been evaluated the most, and therefore the one that appears to have the most promising value across multiple papers. A key limitation of [18F]FMISO as a hypoxia PET tracer relates to its intrinsic pharmacokinetic properties. In particular, its slow clearance from normoxic tissues results in a relatively modest tumor-to-background ratio, which can reduce image contrast and make lesion delineation less precise, especially in heterogeneous tumors such as gliomas [52]. Moreover, long uptake times are required for [18F]FMISO imaging [52]. In addition, this limited contrast may lead to underestimation of small or spatially restricted hypoxic regions, thereby affecting the sensitivity of the technique in capturing intratumoral heterogeneity. Finally, the limited availability of [18F]FMISO due to the need for specialized radiochemistry facilities further restricts its widespread clinical use, confining it mainly to research settings. These factors should be considered when interpreting study results and underscore the need for improved or alternative hypoxia imaging tracers.

## 5. Conclusions

The available evidence supports the possible role of [18F]FMISO PET as a biologically meaningful imaging modality in gliomas. Insights on its value in tumor grading, prognostication, and treatment monitoring, particularly in glioblastoma have been reported; however, the current level of evidence is based mostly on single-center, retrospective and small-sample studies. Moreover, the heterogeneity in study designs, patient populations, and analytical methods are major limits to the drawing of definitive conclusions on the added value of [18F]FMISO imaging in the assessment of gliomas. Future research should focus on standardized imaging protocols, integration with molecular classification frameworks, and validation of hypoxia-guided therapeutic strategies. In this evolving landscape, multiparametric imaging approaches combining hypoxia, metabolism, proliferation, and perfusion may represent the most promising avenues toward the personalized management of gliomas.

## Figures and Tables

**Table 2:** Characteristics of the studies considered for the review.

First Author [N. Ref.]	Year	Country	Study Design	Setting	N. Glioma Pts. (Glioblastoma)
Valk PE [53]	1992	USA	Retrospective	Pretherapy	3 (2)
Bruehlmeier M [54]	2004	Switzerland	Retrospective	Pre- and posttherapy	9 (7)
Rajendran JG [55]	2004	USA, Ireland	Retrospective	Pretherapy	5 (5)
Cher LM [56]	2006	Australia	Prospective	Pretherapy	17 (7)
Spence AM [57]	2008	USA, Ireland	Retrospective	Pre- and posttherapy	22 (22)
Swanson KR [58]	2009	USA	Retrospective	Pre- and posttherapy	24 (24)
Szeto MD [59]	2009	USA	Retrospective	Pretherapy	11 (11)
Kawai N [60]	2011	Japan	Prospective	Pretherapy	10 (10)
Hirata K [61]	2012	Japan	Prospective	Pretherapy	23 (14)
Yamamoto Y [62]	2012	Japan	Retrospective	Pretherapy	30 (16)
Kawai N [63]	2014	Japan, China	Retrospective	Pre- and posttherapy	32 (20)
Barajas RFJ [64]	2016	USA	Prospective	Pre- and posttherapy	4 (0)
Gerstner ER [65]	2016	USA	Prospective	Pretherapy	50 (50)
Yamaguchi S [66]	2016	Japan	Retrospective	Posttherapy	18 (12)
Bekaert L [67]	2016	France	Prospective	Pretherapy	33 (24)
Toyonaga T [84]	2016	Japan	Retrospective	Pretherapy	25 (17)
Toyonaga T [85]	2017	Japan	Retrospective	Pretherapy	32 (32)
Ferreira Da Ponte K [68]	2017	France	Prospective	Pretherapy	23 (23)
Preibisch C [69]	2017	Germany	Prospective	Pretherapy	12 (11)
Abdo R [70]	2019	Canada, France	Prospective	Pretherapy	9 (9)
Abdo R [71]	2019	Canada, France	Prospective	Pretherapy	9 (9)
Miyake K [72]	2020	Japan	Retrospective	Pretherapy	113 (63)
Muzi M [73]	2020	USA, Ireland	Retrospective	Pre- and posttherapy	72 (69)
Abdo R [74]	2021	Canada, France	Prospective	Pretherapy	9 (9)
Collet S [75]	2021	France	Prospective	Pretherapy	31 (31)
Huang S [76]	2021	USA	Prospective	Posttherapy	33 (33)
Suzuki K [77]	2021	Japan	Retrospective	Pretherapy	87 (58)
Uchinomura S [78]	2022	Japan	Retrospective	Pretherapy	62 (62)
Barajas RFJ [79]	2022	USA	Prospective	Posttherapy	6 (6)
Suzuki T [80]	2023	Japan	Retrospective	Posttherapy	7 (7)
Wang Y [81]	2023	Japan	Prospective	Pretherapy	35 (22)
Okamoto M [82]	2024	Japan	Retrospective	Pretherapy	33 (33)
Nehmeh SA [83]	2025	USA	Prospective	Pre- and posttherapy	8 (8)

N.: number; Ref.: reference; Pts.: patients; USA: United States of America.

**Table 3:** Results and main findings of the studies included in the review.

First Author [N. Ref.]	Device	Tracers	Main Findings
Valk PE [53]	PET	[18F]FMISO	[18F]FMISO was able to detect hypoxia in human gliomas in vivo.
Bruehlmeier M [54]	PET	[18F]FMISO and [15O]H_2_O	Late [18F]FMISO images provided a spatial description of hypoxia, independent of BBB disruption and tumor perfusion.
Rajendran JG [55]	PET	[18F]FMISO and [18F]FDG	No correlation between hypoxia and glucose metabolism was present.
Cher LM [56]	PET	[18F]FMISO and [18F]FDG	[18F]FMISO imaging was able to assess hypoxia and was prognostic for treatment outcomes in the majority of patients.
Spence AM [57]	PET	[18F]FMISO	Volume and intensity of hypoxia before radiotherapy were strongly associated with poorer time to progression and survival.
Swanson KR [58]	PET	[18F]FMISO	Local hypoxia induces an angiogenic response that later becomes visible on contrast-enhanced MRI.
Szeto MD [59]	PET	[18F]FMISO	Biological aggressiveness assessed by serial MRI was linked with hypoxic burden assessed on PET using a novel bio-mathematical model for glioma growth and invasion.
Kawai N [60]	PET	[18F]FMISO and [11C]MET	Viable hypoxic tissue assessed by [18F]FMISO PET is related to neovascularization and aggressiveness by [11C]MET PET.
Hirata K [61]	PET and PET/CT	[18F]FMISO and [18F]FDG	[18F]FMISO PET had the ability to distinguish glioblastoma from lower grade gliomas.
Yamamoto Y [62]	PET/CT	[18F]FMISO	[18F]FMISO PET was a potential tracer to noninvasively assess tumor grading in newly diagnosed gliomas.
Kawai N [63]	PET/CT	[18F]FMISO	Preoperative [18F]FMISO uptake is significantly correlated with the expression of VEGF, however with a large overlap of uptake between high- and low-grade glioma. [18F]FMISO PET appeared to be a suitable prognostic biomarker.
Barajas RFJ [64]	PET/MR	[18F]FMISO	The presence of pseudoprogression was associated with the absence of hypoxia. A reduction in hypoxic volume was observed concurrent with therapy in all patients with recurrent tumor.
Gerstner ER [65]	PET/CT	[18F]FMISO	Increased tumor perfusion, vascular volume, vascular permeability, and hypoxia were negative prognostic markers that could be measured safely and reliably using MRI and [18F]FMISO PET.
Yamaguchi S [66]	PET/CT	[18F]FMISO	Recurrent gliomas with decreasing [18F]FMISO accumulation after short-term bevacizumab application could derive a survival benefit. Change in [18F]FMISO PET appearance could identify resistant gliomas.
Bekaert L [67]	PET/CT	[18F]FMISO	Tumor hypoxia was associated with the expression of hypoxia markers and was related to angiogenesis. [18F]FMISO uptake was a mark of aggressiveness.
Toyonaga T [84]	PET/CT	[18F]FMISO	[18F]FMISO uptake within the lesion indicated the presence of histological micro-necrosis.
Toyonaga T [85]	PET/CT	[18F]FMISO and [18F]FDG	Volumes of tumor positive for both tracers were significant predictors for progression-free survival and overall survival.
Ferreira Da Ponte K [68]	PET/CT	[18F]FMISO	A significant correlation was found between hypoxia and hypervascularization.
Preibisch C [69]	PET	[18F]FMISO	Vascular deoxygenation and tissue hypoxia did not show high spatial correspondence, therefore revealing complementary information.
Abdo R [70]	PET/CT	[18F]FMISO	By comparing TBR and TNR with spectral analysis, moderate correlation at specific thresholding values and imaging times were reported. A time of 90 min was demonstrated to be enough to achieve an acceptable tumor contrast.
Abdo R [71]	PET/CT	[18F]FMISO	Some differences in defining the hypoxic volumes within a tumor when using TBR with the single static imaging protocol, while the compartmental analysis allowed for the determination of the levels of hypoxia within the tumor sub-volumes.
Miyake K [72]	PET/CT	[18F]FMISO, [18F]FDG, [11C]MET, and [18F]FLT	Combined administration of different PET tracers might aid in the preoperative differential diagnosis of gliomas according to the 2016 WHO criteria.
Muzi M [73]	PET/CT	[18F]FMISO	SUVpeak, HV and T/Bmax together with age had a significant predictive value when combined in a multivariate prognostic model. Adding selected radiomic features to this model further increased predictive performance.
Abdo R [74]	PET/CT	[18F]FMISO	Dynamic PET acquisition adds more accuracy and flexibility in locating the hypoxia regions in glioblastoma multiforme tumors.
Collet S [75]	PET/CT	[18F]FMISO and [18F]FLT	Different multiparametric imaging methods had value when used to assess tumor heterogeneity and to define tumor volumes and subvolumes that are likely to be resistant to conventional therapies.
Huang S [76]	PET/CT	[18F]FMISO	High hypoxia volume, enhancing and non-enhancing tumor volumes, and srCBV were all inversely correlated to glioblastoma patient outcomes following bevacizumab failure.
Suzuki K [77]	PET/CT	[18F]FMISO and [18F]FDG	Tumor hypoxia was informative for prediction of the IDH mutation status, though with low accuracy.
Uchinomura S [78]	PET/CT	[18F]FMISO and [18F]FDG	[18F]FMISO PET was as helpful for differentiating PCNSL from glioblastoma as [18F]FDG PET.
Barajas RFJ [79]	PET/MR	[18F]FMISO	[18F]FMISO PET/MRI was able to distinguish recurrent tumor from pseudoprogression at the time of presumed disease progression.
Suzuki T [80]	PET/CT	[18F]FMISO	[18F]FMISO-PET effectively visualized tumor microenviroment oxygenation after neoadjuvant bevacizumab. Increased tracer accumulation at the time of recurrence suggested that PET might be useful for monitoring the duration of therapy efficacy.
Wang Y [81]	PET/CT	[18F]FMISO	[18F]FMISO PET might provide a valuable tool for differentiating between IDH mutation status of 2021 WHO classification grade 3 and 4 adult-type diffuse gliomas.
Okamoto M [82]	PET/CT	[18F]FMISO and [18F]FDG	Glucose-6-phosphatase 3 was affluently expressed in glioblastoma tissues with coincidentally high [18F]FDG and [18F]FMISO accumulation. Further, it might work as a prognostic biomarker of glioblastoma.
Nehmeh SA [83]	PET/CT	[18F]FMISO and [18F]FLT	Dual [18F]FMISO and [18F]FLT PET can provide detailed characterization of tumor microenvironment and the interaction of multiple hallmarks that yield radioresistance.

N.: number; Ref.: reference; PET/CT: positron emission tomography/computed tomography; [18F]FDG: [18F]fluorodesoxyglucose; [18F]FMISO: [18F]fluoromisonidazole; [18F]FLT: [18F]fluorothymidine; [15O]H_2_O: [15O]water; BBB: brain–blood barrier; IDH: isocitrate dehydrogenase; WHO: World Health Organization; MRI: magnetic resonance imaging; srCBV: standardized relative cerebral blood volume; PCNSL: primary central nervous system lymphoma; SUVpeak: peak standardized uptake value; HV: hypoxic volume; T/Bmax: tissue SUVmax/blood SUV ratio; TBR: tumor-to-blood uptake ratio; TNR: tumor-to-normal tissue ratio; VEGF: vascular endothelial grow factor; [11C]MET: [11C]methionine.

## Data Availability

Data supporting the reported results can be found using the public scientific databases cited in the text.

## Supplementary Materials

The following supporting information can be downloaded at https://www.mdpi.com/article/10.3390/diagnostics16091284/s1, Table S1: Characteristics of the studies considered for the review.

## Abbreviations

The following abbreviations are used in this manuscript:

[11C]MET	[11C]methionine
[18F]FDG	[18F]fluorodeoxyglucose
[18F]FLT	[18F]fluoroethyl-L-tyrosine
[18F]FMISO	[18F]fluoromisonidazole
AUC	Area under the curve
BBB	Blood–brain barrier
CBV	Cerebral blood flow
HGG	High grade glioma
hMTV	Volume of [18F]FMISO/[18F]FDG double-positive
hTLG	Product between hMTV and [18F]FDG SUVmean
HV	Hypoxic volume
IDH	Isocitrate dehydrogenase
LGG	Low grade glioma
MRI	Magnetic resonance imaging
OS	Overall survival
PCNSL	Primary central nervous system lymphoma
PET	Positron emission tomography
PFS	Progression-free survival
SUV	Standardized uptake value
T/B	tissue SUVmax/blood SUV ratio
TBR	Tumor-to-blood ratio
TNR	Tumor-to-normal ratio
VEGFR	Vascular endothelial growth factor receptor
WHO	World health organization
