# Supplementary material for: Role of [18F]FMISO PET Imaging for the Evaluation of Gliomas: A Comprehensive Literature Review
Source: Diagnostics (Basel). 2026 Apr 24;16(9):1284. doi: 10.3390/diagnostics16091284 (PMC13163255; doi:10.3390/diagnostics16091284)
Supplement: Supplementary file 1 [file diagnostics-16-01284-s001.zip › diagnostics-4240399-supplementary.pdf]

**Table S1.** Characteristics of the studies considered for the review.

| First Author [N. Ref.] | Reported Mean Activity (MBq) | Uptake Time | Reconstruction Methods |
|---|---|---|---|
| Valk PE [53] | 370 | 2-3 hours | ns |
| Bruehlmeier M [54] | 291 for [18F]FMISO, 500-700 for [15O]H2O | 150-170 minutes | FWHM 5 mm |
| Rajendran JG [55] | 3.7/Kg for [18F]FMISO and [18F]FDG | 120-140 minutes | Hanning filter after scatter correction |
| Cher LM [56] | 18.5/Kg for [18F]FMISO, ns for [18F]FDG | 2 hours | 3D reconstruction |
| Spence AM [57] | 3.7/Kg | 120-140 minutes | Hanning filter after corrections for scatter, singles, and random events |
| Swanson KR [58] | 3.7/Kg | 120-140 minutes | Hanning filter after corrections for scatter, singles, and random events |
| Szeto MD [59] | 3.7/Kg | 120-140 minutes | ns |
| Kawai N [60] | 308 for [11F]FMISO, 246 for [11C]MET | 120-140 minutes | Hanning filter (kernel FWHM 10 mm, cutoff frequency 0.4 cycle/projection element) |
| Hirata K [61] | 400 for [18F]FMISO and [18F]FDG | 4 hours | FBP with a Hann filter of 4 mm full-width at half-maximum |
| Yamamoto Y [62] | 3.7/Kg | 120 minutes | OSEM algorithm |
| Kawai N [63] | 270 | 120 minutes | OSEM algorithm |
| Barajas RFJ [64] | 259 | 90 minutes | Time-of-flight |
| Gerstner ER [65] | 3.7/Kg | 110 minutes | 3D FBP and iterative techniques, filter sizes ranged from 2–6mm, reconstructed image resolution varied from 1–6mm |
| Yamaguchi S [66] | 400 | 4 hours | FBP based method with a Hann filter of 4 mm full-width at half-maximum |
| Bekaert L [67] | 400 | 120 minutes | OSEM 2D algorithm (nine subsets and two iterations) and filtered in 3-D with a Butterworth filter |
| Toyonaga T [84] | 400 | 4 hours | FBP or OSEM with a Hann filter of 4 mm full width at half maximum |

| | | | |
|---|---|---|---|
| Toyonaga T [85] | 413.9 for [18F]FMISO, 323.3 for [18F]FDG | 4 hours | FBP or OSEM with a Hann filter of 4 mm full width at half maximum |
| Ferreira Da Ponte K [68] | 5/Kg | 2 hours | OSEM 2D algorithm (9 subsets and 2 iterations) and filtered in 3 dimensions with a Butterworth filter |
| Preibisch C [69] | 194.6 | 120-140 minutes | OSEM (3 × 21 subsets, 172 × 172 matrix, nominal voxel size 1.67 × 1.67 × 2.03 mm3, Gaussian filter 4 mm) |
| Abdo R [70] | 3.7/Kg | 2 and 3 hours | ns |
| Abdo R [71] | 3.7/Kg | 2, 3 and 4 hours | ns |
| Miyake K [72] | ns | ns | FBP with a Hanning filter (kernel FWHM 10 mm, cutoff frequency 0.4 cycle/projection element) |
| Muzi M [73] | 237 or 259 | 110 minutes | 3D-FBP with corrections for attenuation, scatter, random events, dead time, and sensitivity, post-reconstruction 6-mm Hanning filter. |
| Abdo R [74] | 3.7/Kg | 2 and 3 hours | 3D-OSEM algorithm |
| Collet S [75] | 5/Kg | 2 hours | OSEM 2D algorithm (9 subsets and 2 iterations) and filtered in 3 dimensions with a Butter-worth filter on a 1.95 3 1.95 3 3.27 mm voxel size. |
| Huang S [76] | 3.7/Kg | 120 minutes | 3D iterative OSEM with two iterations and 21 subsets, time of flight, PSF-correction, slice thickness 3 mm, matrix size 128 ×128, in-plane reconstruction pixels size 6.3638 mm×6.3638 mm, and a Gaussian post-reconstruction convolution kernel with full width at half maximum of 5 mm. |
| Suzuki K [77] | 262 for [18F]FMISO, 235 for [18F]FDG | 120 minutes | OSEM) algorithm |
| Uchinomura S [78] | 3.7/Kg for [18F]FMISO and [18F]FDG | 120 minutes | OSEM, incorporating correction with PSF and time-of-flight model (5 |

| | | | |
|---|---|---|---|
| | | | iterations, 21 subsets) using a Gaussian filter. |
| Barajas RFJ [79] | 3.7/Kg | 90 minutes | ns |
| Suzuki T [80] | ns | 120 minutes | OSEM |
| Wang Y [81] | 350-550 | 4 hours | Attenuation correction using CT data, post-filtering at 2 mm FWHM, VUE Point HD (3D OSEM), PSF (subsets, 12; iterations, 4; filter cutoff, 2.0 mm). |
| Okamoto M [82] | 413.9 for [18F]FMISO, 323.3 for [18F]FDG | 4 hours | FBP or OSEM with a Hann filter of 4 mm full width at half maximum |
| Nehmeh SA [83] | 298 for [18F]FMISO, 288 for [18F]FLT | 90 minutes | OSEM algorithm (4 iterations; 21 subsets) with time-of -flight and PSF correction. |

FWHM: Full Width at Half Maximum, PSF: Point Spread Function, OSEM: ordered subset expectation maximization, FBP: filtered back projection, MBq: megabecquerel, PET/CT: positron emission tomography/computed tomography, kg: kilogram, ns: not specified, [18F]FDG: [18F]fluorodesoxyglucose; [18F]FMISO: [18F]fluoromisonidazole; [18F]FLT: [18F]fluorothymidine; [15O]H2O: [15O]water; [11C]MET: [11C]methionine, 3D: three dimension.
